# The Nerves of Taste

**Published:** 1896-09

**Authors:** 


### The Nerves of Taste.

L. von Frankl-Hochwart (*Wiener klin. Wochenschrift*) says the lingual nerve supplies fibres for the sense of taste to the anterior two-thirds of the tongue; these pass entirely or for the greater part into the chorda tympani. Clinical observation of processes situated at the base of the brain proves that these fibers enter the fifth nerve; resection of the Gasserian ganglion very often causes ageusia in the anterior portion of the tongue. It is not known whether these fibers of taste are to be found in the second or third branch of the fifth nerve, and the mode of connection with the facial and chorda tympani is unknown. The glosso-pharyngeus is generally recognized as the nerve of taste to the posterior third of the tongue. In some individuals, however, total destruction of the trigeminal nerve by basal growths, trauma or resection, does not interfere with the sense of taste, and probably the glosso-pharyngeus supplies in these cases the entire tongue with taste fibers. Although repeated clinical observation of cases in which the ninth nerve was destroyed has shown alteration of taste only at the posterior part of the tongue, Popl, in his case of compression of the left glosso-pharyngeus by an aneurism, as demonstrated by the autopsy, without involvement of the fifth, was able to observe considerable disturbance of taste in the anterior part of the tongue as well as total ageusia over the left posterior portion.—*International Med. Magazine.*

